# Urine and cerebrospinal fluid biomarkers for early glioma detection: a systematic review

**DOI:** 10.1186/s41512-026-00230-1

**Published:** 2026-06-04

**Authors:** Craig Paterson, Jinyue Yu, Philippa Davies, Jack Read, Jennifer C Palmer, Sarah Dawson, Julian PT Higgins, Kathreena M Kurian

**Affiliations:** 1 Population Health Sciences, Bristol Medical School, University of Bristol, Bristol, UK; 2 Cancer Research Integrative Cancer Epidemiology Programme, University of Bristol, Bristol, UK; 3 Exeter Medical School, University of Exeter, St Luke’s Campus, Exeter, UK; 4 Brain Tumour Research Centre, Bristol Medical School, University of Bristol, Bristol, UK; 5 University of Bristol Medical School & Bristol North Trust, Southmead Hospital, Southmead Road, Bristol, BS1 0NB UK

## Abstract

**Background:**

Liquid biopsies offer a minimally invasive approach for early glioma detection. However, potential non-blood biomarkers in urine and CSF are under-researched. While urine is easier to sample and has fewer proteins/cells, CSF may yield higher detection rates due to its proximity to the glioma. This systematic review evaluates non-blood liquid biopsy biomarkers (proteins, metabolites, cell-free DNA, and miRNA) in urine and CSF for early glioma detection.

**Methods:**

Three electronic databases (MEDLINE, Embase, and BIOSIS) were searched from inception to September 2024. Studies which assessed urine- or CSF-derived biomarkers as a means of identifying glioma patients were included. The primary outcomes of interest were measures of diagnostic test accuracy (specificity, sensitivity, positive predictive value, negative predictive value and area under the ROC curve). QUADAS-2 was used to assess risk of bias in included studies.

**Results:**

We extracted data from 20 studies (669 glioma patients and 597 controls) which met the inclusion criteria. Five of the included studies investigated urine-derived biomarkers, and 15 investigated CSF-derived biomarkers. Meta-analysis was not possible due to the heterogeneous nature of biomarkers collected, analysis methods, and differences in patient demographics.

**Conclusions:**

Conclusions are limited by the paucity of studies and small sample sizes. These limitations highlight the need for well-designed, adequately powered studies to evaluate the diagnostic utility of urine and CSF for glioma liquid biopsies.

**Graphical abstract:**

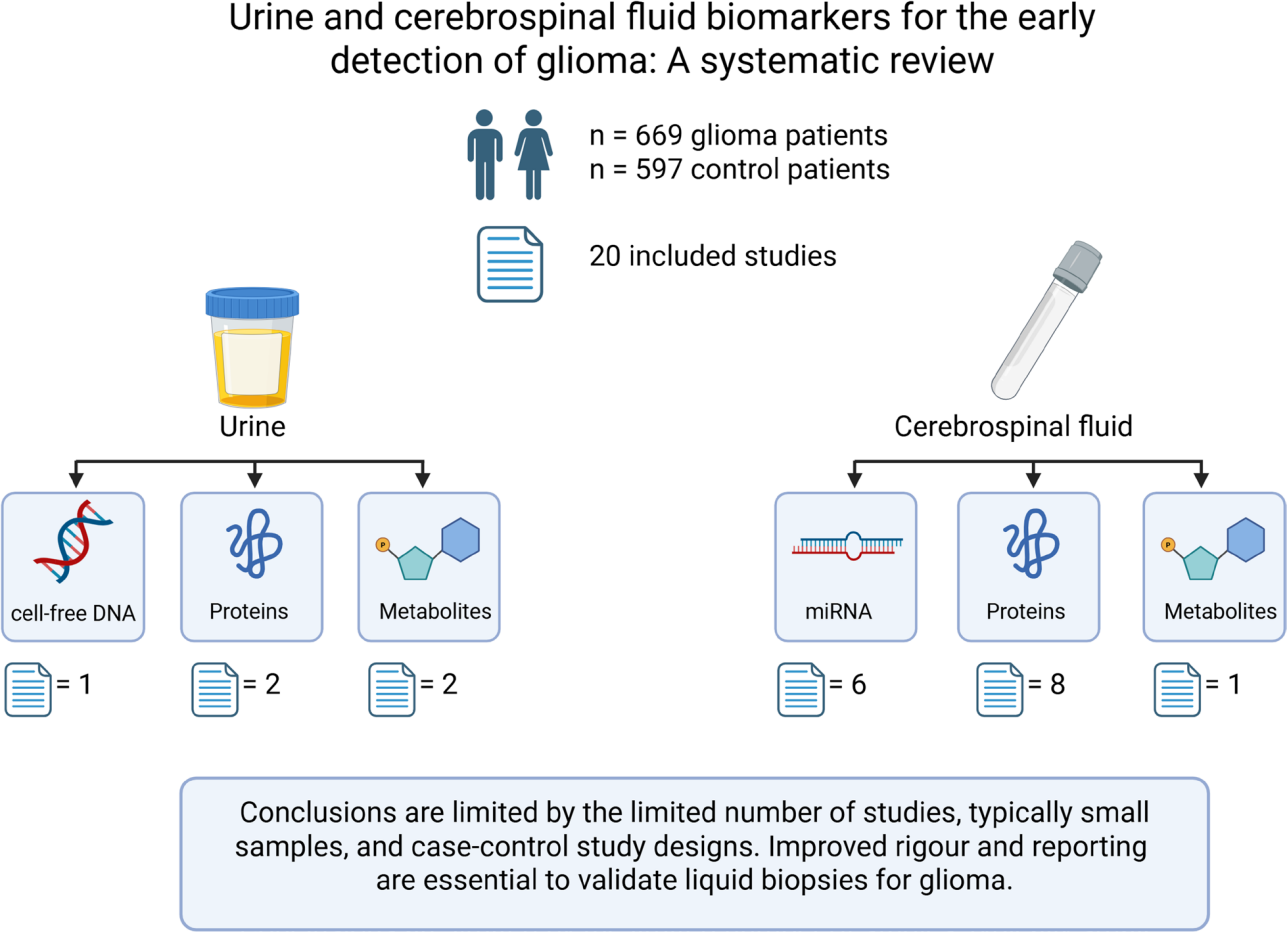

**Supplementary Information:**

The online version contains supplementary material available at 10.1186/s41512-026-00230-1.

## Key points

1) Urine and cerebrospinal fluid are underexplored for glioma liquid biopsy, with typically small, underpowered studies.

2) Multiple biomarkers show promising AUCs but lack precision estimates, representing generally poor reporting.

3) Urine biomarkers show no overlap with CSF or blood; CSF shows overlap with blood but with limited replication.

**Supplementary Information:**

The online version contains supplementary material available at 10.1186/s41512-026-00230-1.

## Introduction

Gliomas account for an average of 20 years of life lost, with a 5-year survival rate of 35.7% [1, 2]. The most common form of glioma, glioblastoma (GBM), has a 5-year survival rate of 6.9% [3, 4]. Therefore, there is an urgent need for earlier detection to improve therapeutic options. Diagnosing glioma can be challenging because patients may present with nonspecific symptoms such as headaches. Currently, imaging (magnetic resonance imaging [MRI]/computed tomography [CT] scans) and biopsy are the gold standard for diagnosis. The protracted diagnostic pathway may impair the ability to deliver effective, timely care and thus contribute to the poor survival rates associated with the disease [3, 4]. Consequently, there is a growing need for other biomarkers that may enable more efficient detection or diagnosis of glioma.

Liquid biopsies offer a potential method that could aid early detection or as an adjunct to triage patients for imaging, yet the potential for this for glioma has not been exploited compared with other cancer types [5, 6]. A recent review by Ali et al., [7] has summarised the existing knowledge of blood-based markers within the context of glioma, examining protein, metabolomic, and cfDNA markers. By contrast, urine and cerebrospinal fluid (CSF) remain relatively under-explored despite their promise as sources of potential biomarkers and their distinct biological and clinical advantages. The relative ease of urine sampling, with evolving ‘omics analyses, may hold valuable insight for the detection of low-abundance biomarkers for the early detection of glioma. By contrast, the proximity of CSF to the brain tumour microenvironment may garner high-abundance biomarkers for glioma which hare more directly reflective of tumour biology. Blood, while an important intermediary biofluid with ease of sampling, was not included in this review because it has been comprehensively evaluated elsewhere [7] . Because our primary objective was to evaluate diagnostic performance, we focused on study designs that could facilitate estimation of diagnostic test accuracy (e.g., through inclusion of appropriate comparator groups), irrespective of whether diagnostic test accuracy metrics were explicitly reported. Consequently, we aimed to systematically review potential urine and CSF biomarkers of glioma and report measures of diagnostic test accuracy where available.

## Methods

The protocol for this systematic review was pre-registered on the International Prospective Register of Systematic Reviews (PROSPERO; CRD42023479231). This systematic review was reported in accordance with PRISMA (Preferred Reporting Items for Systematic Reviews and Meta-Analyses) and SWiM (Synthesis Without Meta-analysis) guidelines. [8, 9] Whilst this systematic review reports measures of diagnostic test accuracy where possible, it is not strictly a systematic review of diagnostic test accuracy studies and, thus, is not reported in line with the PRISMA-DTA guidelines. [10] .

### Data sources and searches

#### Eligibility criteria

To be included in this review, studies must have (i) been cohort, nested case-control, case-control or cross-sectional in design; (ii) reported data related to a non-blood (e.g., cerebrospinal fluid or urine) liquid biopsy biomarker for the early detection or diagnosis of glioma of any grade; and (iii) be patients of any age that were diagnosed with or suspected of having primary or recurrent glioma. Glioma must have been histologically confirmed in line with the WHO grading system before or after liquid biopsy sampling. Finally, studies must have included a minimum of 10 cases and 10 controls. No restriction was put on the patients who served as controls other than that they did not have glioma.

Case studies, conference abstracts, dissertations, and other non-peer-reviewed articles were not eligible for inclusion in this review. Studies that did not report glioma patient data separately (e.g., grouped with other cancers or conditions) or used alternative grading classification systems (e.g., Daumas-Duport) were also excluded.

#### Information sources

Three electronic databases (MEDLINE, Embase, and BIOSIS) were searched from inception until September 2024 with no restrictions applied to language or date of publication. Searches up to November 2023 took a broad approach to cover other systematic reviews [11] ; later searches were targeted for the current review and included named biomarkers identified from earlier searches. Full MEDLINE search strategies for both approaches are available as electronic supplementary material. All search results were uploaded to a web-based review management system (Covidence). Following automatic identification and removal of duplicates, titles and abstracts of remaining records were inspected independently by at least two reviewers, and the full texts of potentially relevant articles were retrieved. Full texts were inspected independently by at least two reviewers, with any discrepancies resolved through consensus or discussion with a third reviewer.

#### Data extraction and risk of bias assessment

Before formal data extraction, four reviewers (CP, JY, PD, and JR) piloted and finalised a data extraction spreadsheet. Subsequently, one reviewer extracted data into the finalised spreadsheet where the accuracy of the extracted data was verified by at least one other reviewer. Bibliographic information, details of the liquid biopsy, and patient and outcome data were extracted for each eligible article. Additionally, diagnostic test accuracy statistics were calculated from published data where possible.

The risk of bias and methodological quality of the included articles was assessed using the QUADAS-2 (Quality Assessment of Diagnostic Accuracy Studies) tool. [12] In line with QUADAS-2 guidance, formal assessments were preceded by a pilot stage conducted by four reviewers (CP, JY, PD, and JR) with input from two principal investigators. As not all included articles were diagnostic accuracy studies (for which the QUADAS is ideally suited), the piloting phase allowed for discussion and agreement between the review team to ensure congruency in their assessments. For Domain 3 (Reference Standard), it was agreed that a full diagnosis of glioma via imaging (MRI/CT scan) and histological confirmation should be the criteria for the reference standard. However, for Domain 4 (Flow and Timing), the criteria for a reference standard were relaxed to a minimum of an MRI and/or CT scan for controls. As patients exhibiting symptoms consistent with a brain tumour will typically undergo neuroimaging as a means of triaging them to appropriate treatments, it was deemed unreasonable to hold patients where histological biopsy was contraindicated (e.g., encephalitis) to the same full reference standard as those with glioma.

#### Data synthesis

Due to the limited number of studies and the substantial differences in the biological signals or pathways being examined by each biomarker, meta-analysis was deemed inappropriate. Consequently, we have summarised descriptive and diagnostic test accuracy statistics (area under the receiver operating characteristic curve [AUC] with corresponding 95% confidence intervals [CI] where reported, sensitivity [Sens], specificity [Spec], positive predictive value [PPV], and negative predictive value [NPV]), in tabular form and narrative synthesis. Where diagnostic test accuracy statistics were not presented in the original article, but relevant data were provided, estimates of Sens, Spec, PPV, and NPV were made using previously published methods. [13] Results are grouped first by liquid biopsy type (i.e., CSF or urine). Results from studies that investigated CSF-derived biomarkers are then grouped by biomarker type (i.e., microRNAs [miRNA] and proteins). Due to the limited number of studies, results from studies that investigated urine-derived biomarkers are presented alphabetically.

## Results

### Literature search and trial selection

**Fig. 1:**
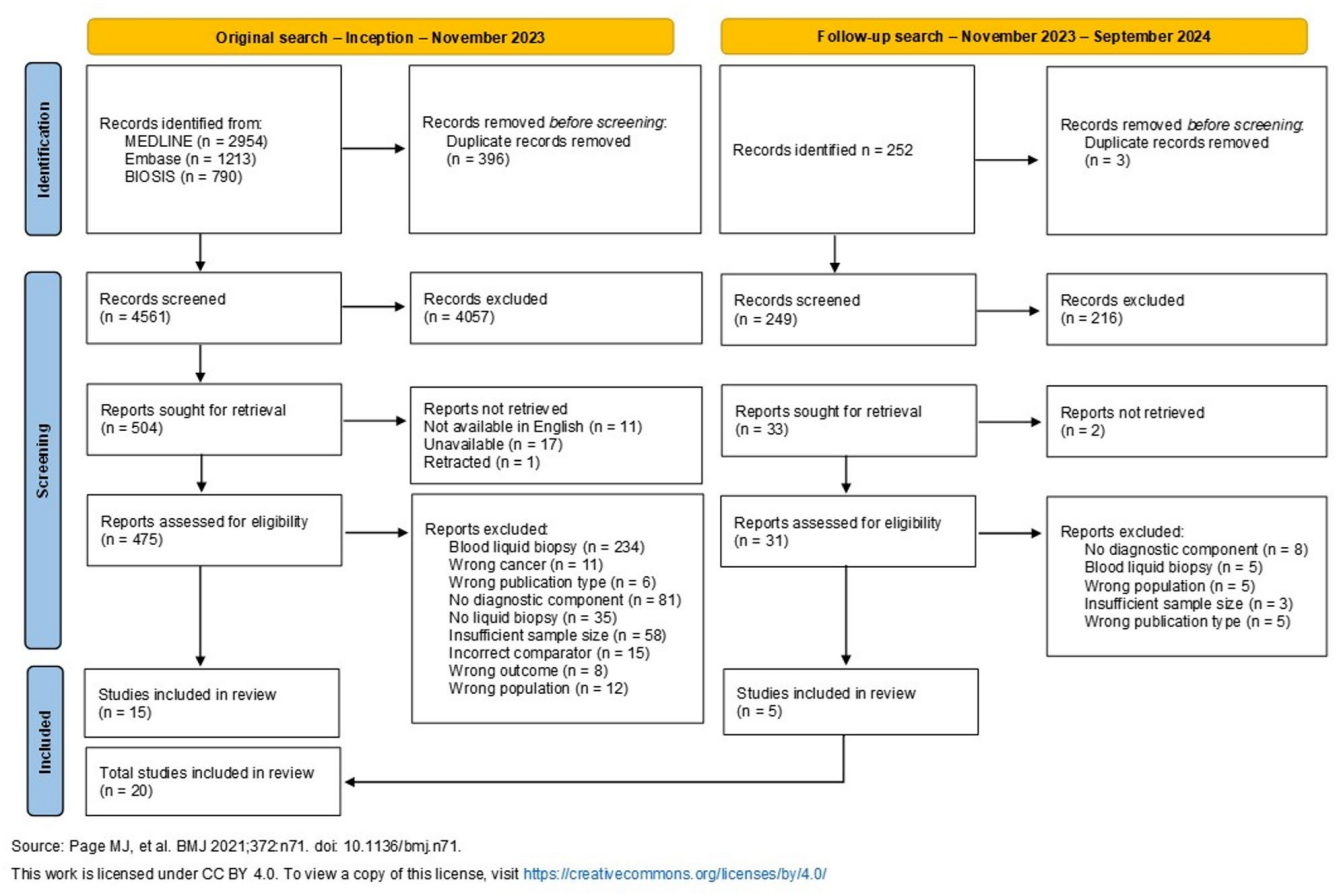
PRISMA flow chart of literature search process

Full details of the literature search process are presented in Fig. 1. In total, 5209 records were identified across all database searches. Following deduplication and title and abstract screening, 506 full texts were screened for eligibility. Of these, 20 met the inclusion criteria for this review. Five of the final 20 studies examined biomarkers found in urine, and the remaining 15 examined biomarkers in CSF.

### Study characteristics

Study characteristics and outcomes are summarised in Table 1. The 20 included studies involved 669 glioma patients and 597 controls. Of the glioma patients, patients were diagnosed as CNS WHO Grade 1 (*n* = 6), CNS WHO Grade 2 (48), CNS WHO Grade 3 (50), and CNS Grade 4 (489). Three papers reported patient diagnoses grouped as low grade (1–2) (19) and high grade (3–4) (57). Of the control participants, patients with a variety of conditions were enrolled. These included patients diagnosed with primary central nervous system lymphoma (PCNSL) (51), brain metastases (43), hydrocephalus (73), unruptured intracranial aneurysm (*n* = 35), neurological disorders (28), meningioma (16), moyamoya disease (6), idiopathic intracranial hypertension (3), vestibular schwannoma (2), intracranial cyst (2), amyloid angiopathy (1), cerebral infection (1), cavernous malformation (1), and controls described as ‘non-oncological’, ‘healthy’, ‘with no known CNS neoplasm’ (335).

**Table 1:** Characteristics of included studies

Author	Country	Liquid biopsy	Biomarker(s)	Characteristics of glioma patients and controls/comparator	Difference between patients and controls	DTA statistics	Classification
Urine							
Mouliere 2021 [11]	UK	Urine	cfDNA fragmentation patterns in urine via WGS. Fragmentation patterns defined by nine bins of fragment length, (30–60, 61–90, 91–120, 121–150, 151–180. 181–210, 211–240, 241–270, and 271–300 bp)	CNS WHO grade (g)1–2:5; g3-4: 30 26 healthy controls 27 patients with non-malignant brain disorders (aneurysm:, radiculopathy: 7, myelopathy: 4, hydrocephalus: 3, arachnoid cyst: 1, cavernoma: 1, hemifacial spasm: 1, Parkinson’s disease: 1)	Glioma-derived cfDNA significantly more fragmented in glioma patients compared to both control groups.	The proportion of fragments between 30 and 60 bp in length shown to have potential diagnostic capability: AUC = 0.885 Classification as cancer vs. non-cancer were explored using logistic regression (LR), random forest (RF), support vector machine (SVM), and binomial generalized linear model with elastic-net regularization (GLMEN): LR: AUC = 0.90 RF: AUC = 0.91 SVM: AUC = 0.80 GLMEN: AUC = 0.91	CfDNA fragment length
Tandle 2013 [9]	USA	Urine	Metabolomic profiling via mass spectroscopy. 368 named metabolites identified across groups	CNS WHO g4: 39 38 healthy controls	46 metabolites that differed significantly in patients vs. controls	Using a random forest analysis, the 46 metabolites demonstrated potential diagnostic capability Sens = 74.4% Spec = 79% PPV = 78.4% NPV = 75%	Metabolites
Shi 2021 [10]	China	Urine	Metabolomic profiling via mass spectroscopy. Total number of metabolites identified is not reported.	Cohort 1: g1:2; g2: 14; g3:12; g4: 32 Cohort 2: g1: 2; g2: 11; g3: 11; g4: 16 100 age and gender-matched healthy controls	61 metabolites in urine were differentially expressed in glioma patients vs. healthy controls. 22 differentially expressed metabolites were found in both urine and plasma	Three metabolites shown to have potential diagnostic capability Histidine – AUC = 0.91–5.96 Glyceric acid – AUC = 0.86–2.92 Uric acid – AUC = 0.82–2.02	Metabolites: Histidine: essential amino acid, multifunctional can act as a neurotransmitter Glyceric acid: organic acid: gluconeogenesis Uric Acid: Purine metabolism
Wu 2020 [8]	China	Urine	Proteomic profiling via mass spectroscopy. Identified 1377 proteins.	Cohort 1: g2: 1; g4: 4 Cohort 2: g2: 2; g3: 3; g4: 6 Meningioma patients: 5; Moyamoya disease patients: 6; Healthy controls; 12	9 urinary proteins showed significant differences between glioma patients and healthy controls – TSP4, MDHM, RINI, CALE, TENA, LEG1, AACT, AHSG, and GELS 7 urinary proteins significantly different between glioma and meningioma patients – AMP4, CD276, LAMP1, NAPSA, LEG1, DNAS1, and BGAL Panel consisting of AACT, TSP4, MDHM, CALR, LEG1, AND AHSG - Best performance AUC − 0.958 - Sens 0.900, Spec 0.917 (no threshold reported)	ROC analyses compared glioma and healthy controls (AUC detailed below) TSP4, MDHM, RINI, CALR, TENA, LEG1, AACT, AHSG and GELS: AUC = 0.927 TSP4, MDHM, RINI, CALR, TENA, LEG1, AHSG and GELS: AUC = 0.923 TSP4, MDHM, RINI, CALR, TENA, LEG1, AHSG: AUC = 0.929 TSP4, MDHM, CALR, TENA, LEG1, AHSG: AUC = 0.939 TSP4, CALR, TENA, AHSG: AUC = 0.921 TSP4, CALR, LEG1, AHSG: AUC = 0.926 TSP4, CALR, LEG1, AHSG, MDHM: AUC = 0.943 TSP4, CALR, LEG1, AHSG, MDHM, AACT: AUC = 0.958	Protein: SP4 (Specificity Protein 4) Type: Transcription factor MDHM (Methylmalonyl-CoA Mutase) Type: Enzyme fatty acid metabolism /Krebs cycle. RINI (Ribosomal Inhibitory Protein) Type: Protein: Protein synthesis CALR (Calreticulin) Type: Chaperone protein, protein folding TENA (Tenascin-A) Type: Extracellular matrix protein LEG1 (Legumain) Type: Cysteine protease enzyme: Legumain is involved in protein Degradation AACT (Alpha-1-Antichymotrypsin) Type: Serine protease inhibitor (serpin) Function: AACT inhibits chymotrypsin-like enzymes, playing a role in modulating protease activity during inflammation and immune response. AHSG (Alpha-2-HS-Glycoprotein) Type: Acute-phase protein: AHSG is involved in mineral metabolism, It also has roles in inflammation and immune response. GELS (Gelsolin) Type: Actin-binding protein Function: Gelsolin regulates actin filament dynamics by severing and capping actin filaments, thus controlling cytoskeletal organization and cell movement. Gelsolin is involved in cell migration, wound healing, and neuroplasticity.
Hallal 2024 [24]	Australia	Urine	Proteomic profiling via mass spectroscopy. 1545 proteins identified across both groups.	g4: 24 14 age and gender-matched healthy controls	31 were differentially expressed proteins between groups (Benjamini-Hochberg adjusted *p* < 0.05)	ROC analyses for 9 proteins at FC *≥* 2: KRT19: AUC = 0.929 RPS2: AUC = 0.948 IST1: AUC = 0.958 RPL18: AUC = 0.912 RPL28: AUC = 0.912 CSTB: AUC = 0.941 ALDH3B1: AUC = 0.912 RPL7A: AUC = 0.906 GNAI2: AUC = 0.920 ROC analysis for a combination of 5 (KRT19, RPS2, RPL18, RPL28, RPL7A): AUC = 0.958	Protein KRT19 Cytoskeletal protein (Intermediate filament) RPS2:Ribosomal protein (40 S subunit) IST1:Cell division and membrane trafficking protein RPL18:Ribosomal protein (60 S subunit) RPL28:Ribosomal protein (60 S subunit) CSTB: Protease inhibitor (serpin family) ALDH3B1: Enzyme (Aldehyde dehydrogenase) RPL7A: Ribosomal protein (60 S subunit) GNAI2: G-protein subunit (Gα inhibitory subunit)
Cerebrospinal fluid							
miRNA							
Akers 2013 [25]	USA	CSF (Ventricular/ lumbar drain or cisternal aspiration at time of craniotomy)	miR-21 via qRT-PCR	Cohort 1 – g4: 14 Cohort 2 – g4: 15 Cohort 1–14 non-oncological patients Cohort 2–14 non-oncological patients	miR-21 significantly elevated vs. control (*p* < 0.0001)	Cohort 1 – Sens = 85%, Spec = 100%, PPV = 100%, NPV = 93%, Cohort 2 – Sens = 87%, Spec = 93%, PPV = 87%, NPV = 86%	MiRNA
Baraniskin 2012 [33]	Germany	CSF (diagnostic lumbar puncture [2 glioma samples collected intraoperatively])	miR-15a miR-15b miR-16 miR-19b miR-21 miR-24 miR-92 miR-106b miR-155 miR-204 via qRT-PCR	g2: 3; g3: 2; g4: 5 Neurological Disorder (ND): 10; Primary Central Nervous System Lymphoma (PCNSL): 23; Brain metastasis (BM): 7	Diff between 10 glioma vs. 10 ND miR-15a - *p* = 0.07 miR-15b - *p* < 0.001 miR-16 - *p* = 0.12 miR-19b - *p* = 0.08 miR-21 - *p* < 0.001 miR-24 - *p* = 0.08 miR-92 - *p* = 0.08 miR-106b - *p* = 0.62 miR-155 - *p* = 0.81 miR-204 - *p* = 0.26	ROC for miR-15b (glioma vs. ND, PCNSL, BM) Sens = 90% Spec = 94.9% AUC = 0.96 Decision tree using miR-15b and miR-21 – Sens = 90%, Spec = 100%, PPV = 100, NPV = 97.6%	MiRNA
Kopkova 2019 [32]	Czechia	CSF (lumbar puncture)	miRNA panel via qRT-PCR	Cohort 1 g1,2: 14; g4: 32 Cohort 2 : 41 Cohort 1–19 hydrocephalus patients Cohort 2–21 hydrocephalus patients	Identified 25 miRNAs significantly different between GBM and control Identified 14 miRNAs significantly different between LGG and control	NR	MiRNA
Qu 2016 [31]	China	CSF (unclear)	miR-21 via qRT-PCR	g2: 9; g3:11; g4: 15 10 controls	miR-21 significantly elevated in patients vs. controls (*p* = 0.004)	AUC = 0.81 (95% CI: 0.68, 0.93)	MiRNA
Teplyuk 2012 [34]	USA and Germany	CSF (lumbar puncture or Ommaya reservoir)	miR-10b miR-21 via qRT-PCR	g4: 19 15 non-neoplastic neurological conditions	miR-10b and miR-21significantly elevated in patients vs. controls (both *p* < 0.001)	SVM modelling using 7 miRNAs (miR-10b, miR-21, miR-125b, miR-141. miR-200a, miR-200b, and miR-200c) – correctly identified 91.2% of samples as GBM or control	MiRNA
Akers 2017 [21]	USA and Germany	CSF (lumbar puncture or cisternal aspiration)	miRNA panel via qRT-PCR miR-21 miR-218 miR-193b miR-331 miR-374a miR-548c miR-520f miR-27b miR-130b	Cohort 1 – g4: 24 Cohort 2 – g4: 40 Cohort 3 – g4: 13 Cohort 4 – g4: 10 Cohort 5 – g4: 18 Cohort 1–15 Cohort 2–27 Cohort 3–19 Cohort 4–12 Cohort 5–20 Normal/non-oncological controls	NR	Cohort 4 (cisternal CSF) – Sens = 80%, Spec = 67%, PPV = 67%, NPV = 80%, AUC = 0.75 (95% CI: 0.53, 0.97) Cohort 5 (lumbar CSF) – Sens = 28%, Spec = 95%, PPV = 84%, NPV = 59%, AUC = 0.83 (95% CI: 0.69, 0.96)	MiRNA
Proteins							
Peles 2004 [12]	Israel	CSF (subarachnoid space or ventricular catheter)	bFGF, VEGF via ELISA	g3: 4; g4: 15 10 communicating hydrocephalus	bFGF and VEGF significantly elevated in patients vs. control (*p* < 0.005)	NR	Protein Growth factor
Sampath 2004 [13]	USA	CSF (lumbar puncture or ventricular drain)	VEGF via ELISA	g3-4: 27 14 no known CNS neoplasm	VEGF significantly elevated in HGG vs. controls (*p* = 0.00003). Important to note that VEGF was undetectable in 12/14 controls	NR	Protein Growth Factor
Koper 2018 [15]	Poland	CSF (subarachnoid space)	Nogo-A (myelin associated inhibitory protein) via ELISA	g1: 1; g2:2; g3: 2, g4:15 11 non-tumoural patients with unruptured intracranial aneurysm	Nogo-A significantly lower in glioma patients vs. controls (median Nogo-A, 359 pg/mL [IQR 276–499] vs. 3452 pg/mL [IQR 1471–5000], respectively	NR	Protein membrane- associated protein, myelin -associated inhibitory protein
Koper 2018 [14]	Poland	CSF (subarachnoid space)	IL-8; CCL2; ICAM-1 via ELISA	g1: 1; g2:2; g3: 2; g4: 15 20 non-tumoural patients with unruptured intracranial aneurysm	IL-8 significantly elevated in patients vs. controls (*p* < 0.001) No sig diff for CCL2 or ICAM-1	IL-8 : AUC = 0.86, Sens = 80%, Spec = 80%, PPV = 80%, NPV = 80%	Protein IL8: Cytokine: Interleukin CCL2: Cytokine: chemokine ICAM1: Cytokine: intercellular adhesion molecule immunoglobulin superfamily
Koper-Lenkiewicz 2021 [16]	Poland	CSF (subarachnoid space)	Nogo-A MAG, OMgp via ELISA	g1: 1; g2: 2; g3: 3; g4: 27 24 non-tumoural patients with unruptured intracranial aneurysm	Nogo-A significantly lower in patients vs. controls (*p* < 0.05) MAG not significantly different OMgp below sensitivity of assay	Nogo-A – Sens = 73%, Spec = 83%, PPV = 86%, NPV = 69%, AUC = 0.87 (Standard error = 0.045)	Protein Nogo A membrane associated protein, myelin associated inhibitory protein MAG: myelin associated glycoprotein: immunoglobulin superfamily Oligodendrocyte-Myelin Glycoprotein Myelin associate protein- inhibits neurite growth
Koper-Lenkiewicz 2019 [17]	Poland	CSF (subarachnoid space)	Neudesin via ELISA	g1: 1; g2:2; g3: 2; g4: 15 11 non-tumoural patients with unruptured intracranial aneurysm	Neudesin concentration greater in patients vs. controls (*p* = 0.046)	NR	Protein: Neuron-Derived Neurotrophic Factor (NENF), neudesin, Neurotrophic factor Atypical growth factor (not a chemokine or traditional cytokine)
Mikolajewicz 2022 [18]	Canada	CSF (intracranially-implanted reservoirs)	Proteomic panel via mass spectroscopy. Identified 755 proteins for analysis.	g4: 22 BM: 17 PCNSL: 14 Hydrocephalus: 20	GAP43 examined as a uniplex classifier for GBM specifically.	GAP43 uniplex - AUC − 0.69 GAP43, TFF3, and CACNA2D2 as a triplex classifier AUC − 0.75 Uniplex and triplex classifier used on subset of IDH-wt GBM (16/22 participants) AUC = 0.68 (95% CI: 0.47, 0.87) AUC = 0.75 (95% CI: 0.58, 0.95)	Protein GAP 43 Growth-Associated Protein 43, promotes neuronal growth, membrane associated phosphoprotein
Landry 2024 [19]	Canada	CSF	Proteomic profiling and DNA methylation profiling via mass spectroscopy. Input data for models included 505,027 unique methylation probes and 1368 unique proteins	g4:20 BM: 17 PCNSL: 14	Not reported (NR)	Three modelling approaches: Model 1 - two platforms (methylome and proteome) to create single classifier Model 2 - same as above plus penalty factor to create datatype specific regularisation parameters Model 3 - separate classifiers for each platform trained on each datatype and posterior probabilities used to train a second layer integrated classifier AUCs (95% CI) in the “GBM vs other” models Model 1 = 0.88 (0.83–0.90), Model 2 = 0.85 (0.83–0.90), Model 3 = 0.89 (0.85–0.90). Thresholds not specified	Protein And/or Epigenetic classifier based on methylation at CpG islands
Möhn 2024 [20]	Germany	CSF	Metabolomic profiling via mass spectroscopy. 630 metabolites detected.	g2:4; g3:2; g4:23 20 non-tumoural controls	Six metabolites showed a significantly greater concentration in glioma group compared to controls: Putrescine (*p* < 0.001) Betaine *p* < 0.001) Xanthine (*p* = 0.002) Glutamate (*p* = 0.002) Acetylcarnitine (*p* = 0.002) Aspartate (*p* = 0.002)	AUC Putrescine: 0.785 Xanthine: 0.762 Betaine: 0.759 Glutamate: 0.757 Aspartate: 0.747 Acetylcarnitine: 0.728	Metabolite: Putrescine- polyamine synthesis- ion channels NMDA receptors Betaine: methionine cycle: Xanthine: Purine base/metabolite Glutamate Full name: L-Glutamic acid: Amino acid, neurotransmitter, Acetylcarnitine: Fatty acid metabolism

Abbreviations: *DTA* diagnostic test accuracy, *NR* not reported, *CSF* cerebrospinal fluid, *cfDNA* cell-free DNA, *miR* microRNA, *qRT-PCR* quantitative reverse transcription polymerase chain reaction, *g1* grade 1, *g2* grade 2, *g3* grade 3, *g4* grade 4, *PCNSL* primary central nervous system lymphoma, *BM* brain metastasis, *ELISA* enzyme linked immunosorbent assay, *IL-8* interleukin-8, *CCL2* chemokine ligand 2, *ICAM-1* intercellular adhesion molecule 1, *ND* neurological disorders, *AUC* area under the curve, *Sens* sensitivity, *Spec* specificity, *PPV* positive predicted value, *NPV* negative predictive value, *GBM* glioblastoma, *LGG* low-grade glioma, *bFGF* basic fibroblast growth factor, *VEGF* vascular endothelial growth factor, *CNS* central nervous system, *Nogo-A* neurite growth isoform A of reiculon-4, *MAG* myelin-associated glycoprotein, *OMgp* oligodendrocyte myelin glycoprotein, *WGS* whole genome sequencing, *cfDNA* cell-freeDNA, *SVM* support vector machine, *FC* fold change

The age of patients with glioma ranged from 15 to 92 years, while the control group ranged from 23 to 70 years. However, eight studies did report the age of controls, five of the included studies did not report the sex of glioma patients, and eight studies did not report the sex of control patients.

### Urine

Table 1 summarizes the diagnostic performance of liquid biopsy biomarkers in urine and cerebrospinal fluid. Five studies reported glioma biomarkers derived from urine collected midstream. [14–18].

### Urinary proteins

Hallal et al., [14] identified 31 differentially expressed proteins between 14 healthy controls and 24 pre-operative CNS WHO grade 4 GBM patients. Of these, nine proteins demonstrated good diagnostic capability individually (AUC *≥* 0.9) with a separate panel of five proteins (KRT19, RPS2, RPL18, RPL28, RPL7A) showing the greatest diagnostic capability (AUC = 0.958).

Wu et al., [15] identified nine proteins (TSP4, MDHM, RINI, CALE, TENA, LEG1, AACT, AHSG, and GELS) that differed significantly between glioma patients and healthy controls and seven proteins (AMP4, CD276, LAMP1, NAPSA, LEG1, DNAS1, and BGAL) that differed significantly between glioma and meningioma patients. Notably, a protein panel consisting of AACT, TSP4, MDHM, CALR, LEG1, and AHSG showed the greatest ability to distinguish between glioma patients and healthy controls (AUC: 0.96, Sens: 90%, Spec: 92%).

### Urinary metabolome

Two studies used metabolomic profiling to identify potential urinary biomarkers. Tandle et al., [16] used metabolomic profiling and identified 46 metabolites that differed significantly between 39 GBM patients and 38 healthy controls. Subsequent random forest analysis using the 46 metabolites demonstrated that they may be able to distinguish between GBM patients and healthy controls (Sens: 74%, Spec: 79%, PPV: 78%, NPV: 75%). Shi et al., [17] identified 61 metabolites that were differentially expressed between glioma patients and healthy controls. Of note, 22 of those metabolites were also differentially expressed in patient plasma. Of the 61 identified metabolites, three were shown to have potential diagnostic capability, histidine (AUC = 0.91), glyceric acid (AUC = 0.86), and uric acid (AUC = 0.82).

### Urinary cfDNA

One study used cfDNA to identify potential urinary biomarkers. Mouliere et al., [18] investigated differences in glioma-derived cell-free DNA fragmentation patterns between glioma patients (LGG and HGG) and controls. Mouliere et al., identified that cfDNA in the urine of HGG and LGG patients was significantly shorter and more fragmented than that of healthy controls and those with non-malignant brain disorders (detailed in Table 1). Additionally, the proportion of fragments between 30 and 60 bp in length was significantly greater in both HGG and LGG groups compared to both control groups and displayed a classification performance between groups (AUC = 0.89).

## CSF

### CSF proteins

#### Vascular endothelial growth factor (VEGF)

Two studies reported outcome data related to VEGF [19, 20]. Peles et al., [19] found that CSF levels of VEGF were significantly higher in HGG patients compared with controls (communicating hydrocephalus patients) (*p* < 0.05). Similarly, Sampath et al., [20] reported that levels of VEGF were significantly elevated in HGG patients compared with non-oncological controls (*p* < 0.05). However, in the case of Sampath et al., [20] VEGF levels were below detectable limits in 86% of control patients. It should be noted that these samples were taken from both lumbar puncture and the ventricular system, whereas Peles et al., [19] sampled from the subarachnoid space or the ventricular system. Of the three patients sampled by lumbar puncture in Sampath et al., one patient had detectable levels of VEGF (0.08 ng), compared with the median VEGF levels of 4.49 ng (range 0.41–17.08 ng) when samples were extracted from the ventricular system.

### Basic fibroblast growth factor (bFGF /FGF2)

Peles et al., [19] found that CSF levels of bFGF were significantly elevated in HGG patients compared to controls with communicating hydrocephalus (*p* < 0.05), although no tests of diagnostic test accuracy were reported.

### Interleukin 8 (IL-8), C-C motif chemokine ligand 2 (CCL2)/ MCP-1 (monocyte chemoattractant protein-1), and intercellular adhesion molecule 1 (ICAM-1)

Koper et al. [21] compared CSF levels of three proteins, IL-8, CCL2, and ICAM-1, between 20 glioma patients and 20 non-tumoural controls with unruptured intracranial aneurysms. Samples were collected during surgery from the subarachnoid space. This study found that CCL2 and ICAM-1 levels were not significantly different between patients and controls. However, IL-8 levels were significantly higher in glioma patients compared to controls (*p* < 0.001) and may be able to distinguish between groups (Sens: 80%, Spec: 80%, PPV: 80%, NPV: 80%).

### Myelin-associated glycoprotein (MAG); oligodendrocyte-myelin glycoprotein (OMgp) neurite outgrowth inhibitor Nogo-A (ANogo-A)

Koper et al., [22] compared CSF levels of Nogo-A between glioma patients and controls with unruptured intracranial aneurysms. Samples were collected during neurosurgery from the subarachnoid space. Results showed that Nogo-A levels were ten-fold higher in controls compared with glioma patients (median [IQR]: 3452 pg/mL [1471–5000] vs. 359 pg/mL [276–499], *p* < 0.001). Later research by the same group compared CSF levels of three myelin-associated proteins, MAG, OMgo, and Nogo-A, between glioma patients and non-tumoural controls with unruptured intracranial aneurysms [23] . Samples were also collected during surgery from the subarachnoid space. This study found that MAG levels were not significantly different between groups (*p* > 0.05) and that OMgp levels were below the detection limit of the assay used. Again, Nogo-A levels were significantly lower in glioma patients compared to controls (*p* < 0.05). This later study showed that Nogo-A may be able to distinguish between groups with limited accuracy (Sens: 73%, Spec: 83%, PPV: 86%, NPV: 69%).

### Neudesin

Koper-Lenkiewicz et al., [24] found that CSF levels of Neudesin were significantly higher in 20 glioma patients compared to 11 non-tumoural controls with unruptured intracranial aneurysms (*p* = 0.046).

### CSF proteomic panel studies

Mikolajewicz et al., [25] performed proteomic panel analysis on the CSF of patients with glioma, PCNSL, brain metastases and hydrocephalus controls. Initially, pairwise differential analyses were performed across all proteins and groups to identify GBM-specific proteins that could be used to construct a GBM classifier. This proteomic classifier was able to distinguish between GBM and other conditions (AUC: 0.95 95% CI [0.80, 1.00], Sens: 89%, Spec: 100%, PPV: 100%, NPV = 92%). Subsequently, using a LASSO approach, individual proteins were identified with the greatest diagnostic potential for each of the target conditions (GBM = GAP43, PCNSL = CACNA2D2, BM = TFF3). Tested individually, GAP43 was able to distinguish between GBM and other groups (AUC = 0.69 95% CI [0.55, 0.84], Sens: 82%, Spec: 60%, PPV: 53%, NPV: 81%). Tested in combination with CACNA2D2 and TFF3, the classifier achieved improved diagnostic test accuracy (AUC: 0.75 95% CI [0.61, 0.89], Sens: 73%, Spec: 87%, PPV: 78%, NPV: 80%). Finally, the individual and combined protein classifiers were tested on a subset of IDH-wt GBM sampled and demonstrated AUCs of 0.68 95% CI [0.47, 0.87] and 0.75 95% CI [0.58, 0.95], respectively.

Landry et al., [26] combined proteome and methylome profiling to create a composite score to distinguish between glioma patients and combined group of brain metastases and primary central nervous system lymphoma patients. This study used three modelling approaches; (1) using both platforms (proteome and methylome) to create a single classifier; (2) model 1 plus a penalty factor to create datatype specific regularisation parameters, and; (3) separate classifiers for each platform, trained on each data type and posterior probabilities to train a second layer integrated classifier. All models showed promising diagnostic potential (AUC [95% CI] model 1 = 0.88 [0.83, 0.90], model 2 = 0.85 [0.83, 0.90], model 3 = 0.89 [0.85–0.90]).

### CSF metabolomics

Möhn et al., [27] performed metabolomic profiling on the CSF of glioma patients and non-tumoural controls. Their results identified six metabolites with significantly greater concentrations in the CSF of glioma patients compared to controls (putrescine, xanthine, betaine, glutamate, aspartate, and acetyl carnitine, all *p* < 0.05). Tested individually, each of these metabolites showed potential diagnostic ability (putrescine, AUC = 0.785; xanthine, AUC = 0.762; betaine, AUC = 0.759; glutamate, AUC = 0.757; aspartate, AUC = 0.747; acetyl carnitine, AUC = 0.728).

### CSF miRNA

MicroRNAs were the most reported CSF-derived biomarkers in the included studies, with 6/12 studies reporting outcomes for either isolated miRNAs or panels of miRNAs.

Four studies reported outcome data related to miR-21 (17–20) making it the most frequently reported individual miRNA across the included studies. All four studies identified that patients (ranging grades CNS WHO grade 2–4) had significantly elevated CSF concentrations of miR-21 compared to controls (all *p* < 0.004). Additionally, two studies reported diagnostic test accuracy statistics (17,19). Akers et al., (17) reported that across their two cohorts of glioblastoma patients (Cohort 1: *n* = 14; Cohort 2: *n* = 15), miR-21 was able to distinguish between patients and non-oncological controls (Cohort 1: *n* = 14; Cohort 2: *n* = 14) (Sens: 85–87%, Spec: 93–100%, PPV: 87–100%, NPV: 86–93%). Separately, Qu et al. reported an AUC of 0.81 (95% CI: 0.68, 0.93) for miR-21, in their sample of 35 glioma patients and ten controls.

In addition to miR-21, Baraniskin et al., (18) reported group differences between patients and controls for CSF concentrations of nine additional miRNAs (miR-15a, miR-15b, miR-16, miR-19b, miR-24, miR-92, miR-106b, miR-155, miR-204). Baraniskin et al., reported that levels of miR-15b were significantly elevated in patients vs. controls (*p* < 0.001), while no further statistically significant differences between groups were reported. Further, Baraniskin et al. tested a two-step classification tree, firstly using miR-15b (Sens: 90%, Spec: 95%, PPV: 82%, NPV: 97%) and secondly incorporating miR-21 (Sens: 90%, Spec: 100%, PPV: 100%, NPV: 98%). Additionally, Tepluk et al., reported that miR-10b was significantly elevated in patients compared to controls (*p* < 0.001). Teplyuk et al., (20) subsequently used a support vector machine learning approach informed by levels of miR-10b, miR-21, miR-125b, miR-141, miR-200a, miR-200b, and miR-200c, to correctly identify 91.2% of samples as either GBM or control.

Two studies reported using panels of miRNAs to distinguish between glioma patients and controls (21,22). Kopkova et al., (21) identified a panel of 25 miRNAs that were significantly different between GBM patients and controls (hydrocephalus patients). Of the 25 miRNAs, 20 were significantly elevated (miR-196a-5p, miR-4306, miR-10a-5p, miR-1255b-5p, miR-549a, miR-10b-5p, miR-196b-5p, miR-199b-3p, let-7b-5p, miR-574-5p, miR-152-3p, miR-1247-3p, miR-944, let-7c-5p, miR-224-5p, miR-335-5p, miR-17-3p, miR-365b-5p, miR-10b-3p, miR-10a-3p) and 5 were significantly lower in GBM patients compared to controls (miR-4791, miR-30c-5p, miR-30e-5p, miR-127-3p, 4454). Additionally, the same study reported a panel of 14 miRNAs that were significantly different between LGG and controls (hydrocephalus patients).

Akers et al., [28] developed a 9 miRNA panel to distinguish GBM patients from non-oncological controls. The panel was developed using cisternal and lumbar CSF derived from three independent cohorts that consisted of 77 GBM patients and 61 non-oncological controls. The panel, which was subsequently tested across two validation cohorts, consisted of miR-21-5p, miR-218-5p, miR-193b-3p, miR-331-3p, miR-374a-5p, miR-548c-3p, miR-520f-3p, miR-27b-3p, miR-30b-3p. In the first validation cohort, consisting of ten GBM patients and 12 non-oncological controls, the panel was tested using cisternal CSF and yielded a sensitivity of 80% and specificity of 67% (PPV: 67% NPV: 80%). Subsequently, the panel was tested using lumbar CSF in a larger cohort of eight GBM patients and 20 non-oncological controls, yielding a sensitivity of 28% and a specificity of 95% (PPV: 83% NPV: 59%).

### Risk of bias

Risk of bias assessments for all included studies are detailed in Table 2. A majority of studies (10/19) had a high risk of bias related to patient selection [14, 19, 21, 22, 24, 26, 27, 29–31], eight were deemed to have an unclear risk of bias [15–17, 20, 25, 28, 32, 33], and one was deemed to be low risk [18] . Nine studies were deemed as low risk of bias for index test [15, 19, 21, 22, 24–26, 28, 31], four were deemed as high risk [20, 29, 32, 33], and six had an unclear risk of bias [14, 16–18, 27, 30]. All studies were evaluated as being at a low risk of bias for reference standard. Related to flow and timing, 13 studies were deemed as low risk of bias [17, 19, 21, 22, 24–29, 31, 32], two were at a high risk of bias [16, 33], and four had an unclear risk of bias [15, 18, 20, 30]. The QUADAS-2 tool also assesses concerns of applicability of the included studies across three domains; patient selection, index test, and reference standard. Related to patient selection, 14 studies were deemed to be of low concern [14–18, 20–22, 24–28, 31], one study was deemed to be high concern [33] , and the remaining four studies were graded as having unclear concerns of applicability [19, 29, 30, 32]. Related to the index test, 13 studies were deemed as low concern [14–19, 21, 22, 26–28, 30, 32], five were deemed as high concern [20, 24, 29, 31, 33], and one study was unclear [25] . Finally, related to reference standard, all studies were deemed to be of low concern.

**Table 2:** Quality assessment and risk of bias assessments for included studies

Study	Risk of bias				Applicability concerns			
	Patient selection	Index test	Reference standard	Flow and timing	Patient selection		Index test	Reference standard
Akers et al., 2013 [29]	High	High	Low	Low	Unclear		High	Low
Akers et al., 2017 [28]	Unclear	Low	Low	Low	Low		Low	Low
Baraniskin et al., 2012 [32]	Unclear	High	Low	Low	Unclear		Low	Low
Hallal et al., 2024 [14]	High	Unclear	Low	Low	Low		Low	Low
Koper et al., 2018 [21]	High	High	Low	Low	Low		Low	Low
Koper et al., 2018 [22]	High	Low	Low	Low	Low		Low	Low
Koper-Lenkiewicz et al., 2019 [24]	High	Low	Low	Low	Low		High	Low
Koper-Lenkiewicz et al., 2021 [23]	High	High	Low	Low	Low		Low	Low
Kopkova et al., 2019 [31]	High	Low	Low	Low	Low		High	Low
Landry et al., 2024 [26]	High	Low	Low	Low	Low		Low	Low
Mikolajewicz et al., 2022 [25]	Unclear	Low	Low	Low	Low		Unclear	Low
Möhn et al., 2024 [27]	High	Unclear	Low	Low	Low		Low	Low
Mouliere et al., 2021 [18]	Low	Unclear	Low	Unclear	Low		Low	Low
Peles et al., 2004 [19]	High	Low	Low	Low	Unclear		Low	Low
Qu et al., 2016 [30]	High	Unclear	Low	Unclear	Unclear		Low	Low
Sampath et al., 2004 [20]	Unclear	High	Low	Unclear	Low		High	Low
Shi et al., 2021 [17]	Unclear	Unclear	Low	Low	Low		Low	Low
Tandle et al., 2013 [16]	Unclear	Unclear	Low	High	Low		Low	Low
Teplyuk et al., 2012 [33]	Unclear	High	Low	High	High		High	Low
Wu et al., 2020 [15]	Unclear	Low	Low	Unclear	Low		Low	Low

## Discussion

To our knowledge, this is the first systematic review of urine and cerebrospinal fluid biomarkers for the early detection of glioma using liquid biopsies.

### Urine-derived biomarkers

Conclusions relating to urinary biomarkers from our study are limited because only five studies met the inclusion criteria, four of which used metabolomic and proteomic techniques and one screened for cell-free DNA length (see Table 1). Moreover, case numbers (< 100) and controls in all the urinary studies are small. Although the AUCs quoted for certain biomarkers are > 0.9 in 4/5 urinary studies, caution in interpretation is required because the sample sizes are low and confidence intervals are not quoted.

Metabolomic and proteomic analyses offer a means of assessing panels hundreds to thousands of potential biomarkers within each sample [15, 16]. Urine may be suited to these types of investigations because it can be collected in large volumes non-invasively with relative ease compared with other liquid biopsies (i.e., blood and CSF) [15, 16]. Urine is relatively free of background protein in healthy individuals, although normal urinary metabolites include nitrogenous, organic acid, carbohydrate, amino acid and hormonal catabolites [15, 16]. Normal urinary cfDNA originates from renal and urinary tract cells via natural cell turnover and systemic cfDNA is filtered through the kidney glomerular filtration [18] . The characteristics of normal urinary cfDNA are short fragments (typically < 200 base pairs), with low concentration in healthy individuals and mostly nuclear and mitochondrial DNA which reflects local (e.g., bladder, kidney) and sometimes systemic biological activity [34, 35].

The main disadvantage with the nature of the urinary protein and metabolic biomarkers highlighted in our study (see Table 1 Classification column) relating to disrupted metabolic pathways in amino acid, carbohydrate and nucleotide metabolism, inflammation and structure, is that they may not be specific to glioma pathology in the brain but may represent a generalised response to disease processes [15, 16]. Previous urinary studies reporting the greatest diagnostic potential typically used large panels of metabolites or proteins using gas/liquid chromatography or mass spectrometry however again sample sizes were small [15, 16]. Several potentially relevant metabolites have been identified in both blood and urine. However, these biomarkers may not be specific to glioma. For example, Shi et al. [17] identified 22 metabolites that were dysregulated in the blood and urine of glioma patients compared to healthy controls. However, histidine was the only metabolite to show acceptable diagnostic capability (AUC > 0.80) across both liquid biopsies. Elevated histidine has previously been associated with other cancers, specifically breast and colorectal cancers [34, 35]. However, histidine is a precursor to the inflammatory marker, histamine, and as such, the elevated histidine levels are unlikely to be a specific marker of any cancer. [36] Similarly, cysteine has been identified in both urine [16] and blood [37] metabolomes of glioma patients. However, cysteine has a complex role in the tumour microenvironment and has been identified as a metabolite of interest in several solid tumours [37] . Our review did not identify urinary protein biomarkers that have also been identified in blood. In blood, protein biomarkers for glioma include GFAP, S100B and neurofilament light chain (NfL) which reflect the blood- brain barrier breakdown or glioma- specific proteins such as EGFR/EGFRvIII [7]. As such, it is unlikely that any single urinary protein or metabolite could serve as a specific marker of glioma and a panel signature approach may be preferable.

Beyond the primary limitation of small sample size in the urinary cfDNA study, an additional challenge is distinguishing potentially low levels of glioma cfDNA from normal cfDNA based on fragment length alone, rather than utilizing mutation-specific cfDNA detection (e.g., IDH1, TERT promoter mutations) [18] . Notably, studies on urinary extracellular vesicles (EVs)/exosomes were excluded from our study due to insufficient case and control numbers. However, literature reports have demonstrated that glioma-derived exosomal RNA or protein (e.g., EGFRvIII, MGMT mRNA) can be detected in urine [14] .

### CSF-derived biomarkers

CSF was the most frequently investigated non-blood liquid biopsy in this review (12/15 studies), although, like urine, the lack of investigations with sufficient cases and controls limits conclusions. CSF has been shown to contain greater concentrations of potential biomarkers for CNS malignancies compared to other liquid biopsies such as blood and urine [38–40]. Of the CSF biomarkers assessed in this review, miRNAs were the most frequently reported (6/12 studies). miRNAs are a class of small non-coding RNAs that play a role in various processes within the body and have garnered increased interest as a potential cancer biomarker. Notably, serum miRNAs have been reported as potential biomarkers for melanoma [41] , non-small cell lung [42] , prostate [43, 44], breast [45] , pancreatic [46] , and colorectal cancers [47] . Similarly, serum levels of certain miRNAs have been associated with glioma cancers, particularly miR-21 [7]. Indeed, miR-21 was the single most reported CSF biomarker in this review, with 4 studies investigating its utility either individually or as a part of a broader panel of miRNAs with measures of diagnostic test accuracy demonstrating acceptable levels of both sensitivity and specificity (Table 1). As noted, however, miR-21 and several other miRNAs identified in studies within this review (miR-10b, miR-24, miR-92, miR-106, miR-130, miR-155, miR-520) have been identified as being pivotal in solid tumour development and proliferation, and are not unique to glioma cancers [48–50]. As such, the utility of single miRNAs as diagnostic markers for glioma cancers specifically may be limited. Instead, future work may focus on identifying a miRNA profile for glioma cancers that could serve as a diagnostic tool.

Aside from the use of miRNAs, the remaining studies (Table 1) assessed CSF-derived biomarkers investigated either levels of single proteins such as growth factors VEGF, bFGF, and interleukin IL-8, myelin-associated, neuronal- associated structural proteins using ELISA or used a panel proteomic, metabolomic or methylome classifier approach. Like urine, there were some promising biomarker results with AUC > 0.9 which has been described, however the low sample sizes and relatively wide confidence intervals or lack of confidence intervals necessitates caution in interpretation.

The ability to draw firm conclusions or make recommendations is limited by some of the inherent difficulties of sampling CSF. One issue is the variety of CSF sampling locations reported in this review. CSF is typically sampled via lumbar puncture or intraoperatively at cisternal or ventricular sites. Evidence suggests that concentrations of certain miRNAs and proteins differ significantly between CSF samples collected via lumbar puncture and ventricular/cisternal CSF [25, 51, 52]. Indeed, as an example in this review, Akers et al., [28] showed that the diagnostic performance of the same miRNA panel had lower sensitivity when collected via lumbar puncture (28%) compared to intraoperatively collected CSF (80%). As such, comparing results from studies which have sampled CSF at different locations may be inappropriate and obfuscate the true utility of any potential biomarker. Related to the utility of CSF-derived biomarkers, it should be noted that lumbar puncture is contraindicated for certain patients with space-occupying lesions with mass effect due to increased intracranial pressure [53] . Further, ventricular and/or cisternal CSF is typically sampled opportunistically during surgery. Consequently, the utility of CSF-derived biomarkers within a diagnostic pathway may be limited to individuals whose tumours are not readily biopsied, whether that be due to the location of the tumour or a patient’s inability to endure surgery.

### Strengths and limitations

Despite the rigorous methods employed in this review, several potential limitations of the literature should be acknowledged. Firstly, a significant limitation of this work is the inability to quantitatively synthesise the findings. However, due to the breadth of biomarkers employed, differences in control patient characteristics, and sites of liquid biopsy collection, quantitative synthesis was deemed inappropriate as it may lead to erroneous conclusions. Despite this, the current review consolidates the existing literature related to non-blood liquid biopsy biomarkers for the early detection and diagnosis of glioma.

Secondly, from a reporting perspective, more than half of all included studies failed to fully report demographic details of patients and/or controls. By failing to report these demographics, the ability to compare findings across studies may be compromised, especially if the presence or abundance of certain biomarkers is likely to significantly differ between sexes or across the lifespan irrespective of disease [54] . Thirdly, from a methodological perspective, all the studies in this review used a two-gate or case-control design; that is, patients with known disease (glioma) and those with no known disease were recruited [55] . Further, a majority (~ 89%) of participants in the reviewed studies were diagnosed with high-grade glioma (i.e., grades 3 and 4). Specific to studies examining CSF-derived biomarkers, the inherent difficulty of sampling CSF lends itself to a two-gate design. Sampling CSF typically requires a lumbar puncture or collection during neurosurgery. As such, it is likely that patients serving as controls in these studies were not necessarily selected based on clinical or physiological reasoning but rather on the criteria that CSF could be sampled from them during their care. This two-gate study design is likely to inflate estimates of diagnostic test accuracy [56, 57] and raises concerns of selection bias and, with it, other unknown confounding which might influence the magnitude of diagnostic test accuracy and the conclusions drawn.

## Conclusion and implications

Urine and cerebrospinal fluid (CSF) are under researched for glioma liquid biopsies with existing studies limited by small, underpowered cohorts and inadequate case-control design highlighting the need for larger cohorts. Urine biomarkers are reported with an AUC > 0.9, but with small samples sizes and lacking confidence intervals, limiting interpretability. There is little overlap with reported blood or CSF biomarkers, therefore the extent to which urine biomarkers detect specific glioma pathology remains unclear. CSF biomarkers are reported with an AUC > 0.9, with small samples sizes and lacking confidence intervals like urine. Certain MiRNAs e.g. mi21 are reported in more than one study. Lack of ease of CSF sampling remains a barrier to clinical translation.

## Supplementary Information

Supplementary Material 1

## Data Availability

No new data were generated or analysed in support of this review.
