# Supplementary material for: Urine and cerebrospinal fluid biomarkers for early glioma detection: a systematic review
Source: Diagn Progn Res. 2026 Jun 4;10:16. doi: 10.1186/s41512-026-00230-1 (PMC13235107; doi:10.1186/s41512-026-00230-1)
Supplement: Supplementary file 1 — Supplementary Material 1 [file 41512_2026_230_MOESM1_ESM.docx]

# Supplementary Materials

Contents

[S1. Original MEDLINE Search Strategy 2](#_Toc199486902)

[S2. Follow-up targeted search strategy 5](#_Toc199486903)

# S1. Original MEDLINE Search Strategy

| Ovid MEDLINE(R) ALL <1946 to November 01, 2023> | | |
| --- | --- | --- |
| 1 | [Gliomas or Brain/CNS Metastases] |  |
| 2 | exp Glioma/ | 100240 |
| 3 | (glioma* or astrocytoma* or astroblastoma* or ependymoma* or subependymoma* or craniopharyngioma* or oligodendroglioma* or glioblastoma* or GBM* or oligoastrocytoma* or xanthoastrocytoma* or ganglioglioma* or gangliocytoma* or gliosarcoma* or glioneuronal* or (glial adj2 (tumor* or tumour* or cancer* or neoplasm*))).mp. | 145505 |
| 4 | (brain adj1 metasta*).tw,kf. | 19319 |
| 5 | ((cereb* or intracereb* or choroid plexus or crani* or intracrani* or intra-crani* or infratentori* or infra-tentori* or subtentori* or sub-tentori* or supratentori* or supra-tentori* or hypothalam* or pituitar*) adj3 metasta*).tw,kf. | 6519 |
| 6 | ((mening* or leptomening*) adj3 metasta*).tw,kf. | 2999 |
| 7 | ((CNS or central nervous system or spinal cord or epidur*) adj3 metasta*).tw,kf. | 4223 |
| 8 | exp *central nervous system neoplasms/ or exp *brain neoplasms/ | 178589 |
| 9 | or/2-8 | 266205 |
| 10 | [Biomarkers-1] |  |
| 11 | ((detect* or diagnos* or monitor* or predict* or prognos* or screen*) adj3 (biomarker* or bio* marker*)).tw,kf. | 115344 |
| 12 | ((detect* or diagnos* or monitor* or predict* or prognos* or screen*) adj3 (tumor* or tumour* or cancer* or neoplas* or metasta*) adj3 (biomarker* or marker* or indicator*)).tw,kf. | 20313 |
| 13 | 11 or 12 | 126427 |
| 14 | 9 and 13 | 3639 |
| 15 | ((detect* or diagnos* or monitor* or predict* or prognos* or screen*) adj3 (glioma* or astrocytoma* or astroblastoma* or ependymoma* or subependymoma* or craniopharyngioma* or oligodendroglioma* or glioblastoma* or GBM* or oligoastrocytoma* or xanthoastrocytoma* or ganglioglioma* or gangliocytoma* or gliosarcoma* or glioneuronal* or glial tumor* or glial tumour* or glial cancer* or glial neoplasm*) adj3 (biomarker* or marker* or indicator*)).tw,kf. | 1235 |
| 16 | ((detect* or diagnos* or monitor* or predict* or prognos* or screen*) adj5 (brain or cereb* or intracereb* or choroid plexus or crani* or intracrani* or intra-crani* or infratentori* or infra-tentori* or subtentori* or sub-tentori* or supratentori* or supra-tentori* or hypothalam* or pituitar* or mening* or leptomening* or CNS or central nervous system or spinal cord or epidur*) adj5 (tumor* or tumour* or cancer* or neoplas* or metasta*) adj5 (biomarker* or marker* or indicator*)).tw,kf. | 375 |
| 17 | 14 or 15 or 16 | 4399 |
| 18 | [Liquid Biopsy] |  |
| 19 | (liquid bio* or bod* fluid* or blood or sera or serum or plasma or cerebrospinal fluid or CSF or urine or urinalys* or circulating or cell free or extracellular or extra-cellular or exosom*).mp. | 6062205 |
| 20 | 17 and 19 | 1080 |
| 21 | exp Central Nervous System Neoplasms/ and Neoplastic Cells, Circulating/ and Biomarkers, Tumor/ | 68 |
| 22 | 20 or 21 | 1143 |
| 23 | [Biomarkers-2] |  |
| 24 | BIOMARKERS, TUMOR/ | 178548 |
| 25 | *BIOMARKERS/ or *BIOLOGICAL MARKER/ | 46516 |
| 26 | (biomarker* or bio* marker*).ti,kf. | 172958 |
| 27 | (biomarker* or bio* marker*).ab. /freq=3 | 60161 |
| 28 | or/24-27 | 366688 |
| 29 | 9 and 28 | 11042 |
| 30 | ((glioma* or astrocytoma* or astroblastoma* or ependymoma* or subependymoma* or craniopharyngioma* or oligodendroglioma* or glioblastoma* or GBM* or oligoastrocytoma* or xanthoastrocytoma* or ganglioglioma* or gangliocytoma* or gliosarcoma* or glioneuronal* or glial tumor* or glial tumour* or glial cancer* or glial neoplasm*) adj3 (biomarker* or marker* or indicator*)).tw,kf. | 2640 |
| 31 | ((brain or cereb* or intracereb* or choroid plexus or crani* or intracrani* or intra-crani* or infratentori* or infra-tentori* or subtentori* or sub-tentori* or supratentori* or supra-tentori* or hypothalam* or pituitar* or mening* or leptomening* or CNS or central nervous system or spinal cord or epidur*) adj5 (tumor* or tumour* or cancer* or neoplas* or metasta*) adj5 (biomarker* or marker* or indicator*)).tw,kf. | 1451 |
| 32 | or/29-31 | 13172 |
| 33 | 19 and 32 | 3308 |
| 34 | ((cell free or circulat* or exosom* or extracellular or extra-cellular) adj3 (microRNA or micro-RNA or miRNA or DNA or protein?)).mp. | 84404 |
| 35 | ((cell free or circulat* or exosom* or extracellular or extra-cellular) adj3 (biomarker* or marker*)).tw,kf. | 11898 |
| 36 | 34 or 35 | 93879 |
| 37 | 9 and 36 | 1513 |
| 38 | 33 or 37 | 4394 |
| 39 | [Diagnostic/Prognostic Filter] |  |
| 40 | Diagnosis/ | 17535 |
| 41 | Diagnosis, Differential/ | 468605 |
| 42 | "Diagnostic Techniques and Procedures"/ | 3730 |
| 43 | Diagnostic Test Approval/ | 112 |
| 44 | Diagnostic Tests, Routine/ | 15192 |
| 45 | Molecular Diagnostic Techniques/ | 13786 |
| 46 | (diagnos* adj3 (analys* or assay* or immunoassay* or classif* or differenti* or method* or kit or kits or panel? or predict* or screen* or system* or technique* or test*)).ab. | 422304 |
| 47 | diagnos*.ti,kf,hw,fx. | 4614809 |
| 48 | (diagnos* adj2 (performance or value?)).ab. | 65817 |
| 49 | (DTA or (diagnos* adj2 accura*)).tw,kf. | 118271 |
| 50 | "sensitivity and specificity"/ or "predictive value of tests"/ or roc curve/ or signal-to-noise ratio/ or "limit of detection"/ | 650037 |
| 51 | (sensitivity or specificity).tw,kf. | 1273978 |
| 52 | likelihood ratio*.tw,kf. | 20129 |
| 53 | (predict* adj4 val*).tw,kf. or predict*.ti. | 618771 |
| 54 | (prognos* adj4 val*).tw,kf. or prognos*.ti. | 253435 |
| 55 | ((re-test or retest or test-retest) adj reliability).tw,kf. | 25022 |
| 56 | ((accura* or reliab* or valid*) and (analys* or assay* or immunoassay* or classif* or detect* or diagnos* or differenti* or predict* or technique* or test*)).tw,kf. | 1843816 |
| 57 | Validation Study/ | 109250 |
| 58 | (validat* or validity).tw,kf. | 926622 |
| 59 | area under curve/ | 45902 |
| 60 | observer variation/ | 45072 |
| 61 | (observer adj variation*).tw,kf. | 1722 |
| 62 | ((degree? or rate* or rating) adj3 agreement?).tw,kf. | 12458 |
| 63 | ((detect* or diagnos*) and agreement?).tw,kf. | 84861 |
| 64 | Receiver Operating Characteristic/ | 71576 |
| 65 | (receiver operating characteristic* or ROC).tw,kf. | 154368 |
| 66 | likelihood functions/ | 23805 |
| 67 | diagnostic error/ or false negative result/ or false positive result/ or missed diagnosis/ or false negative reactions/ or false positive reactions/ | 78773 |
| 68 | (false adj (positiv* or negativ*)).tw,kf. | 91888 |
| 69 | (QUADAS* or STARD).mp. | 3765 |
| 70 | laboratory diagnosis/ | 23916 |
| 71 | (reference standard? or gold standard?).tw,kf. | 115334 |
| 72 | (prognosis or progression or recurrence).hw. | 1056704 |
| 73 | (predict* adj3 (progressi* or recurren* or surviv*)).tw,kf. | 89352 |
| 74 | progression free survival.mp. | 71791 |
| 75 | or/40-74 | 8245063 |
| 76 | 38 and 75 | 2623 |
| 77 | 22 or 76 | 3027 |
| 78 | exp animals/ not humans/ |  |
| 79 | ((animal model* or mouse or mice or murine* or rat or rats or rodent* or muridae or murids or rabbit* or leporine* or leporidae or guineapig* or cavies or caviidae or hamster* or cricetidae or gerbil* or gerbillinae or cat or cats or feline* or felidae or dog or dogs or canine* or canidae or pig or pigs or piglet* or minipig* or swine* or porcine* or suidae or horse or horses or donkey or donkies or burros or asses or equine* or equidae or sheep or lamb or lambs or ovine or ovidae or goat or goats or cow or cows or cattle or bovine* or bovidae or primate* or monkey or monkeys or macaque or macaques or marmoset or marmosets) not human*).ti. | 2438431 |
| 80 | 78 or 79 | 5560178 |
| 8 | 77 not 80 | 2954 |

# S2. Follow-up targeted search strategy

|  | Ovid MEDLINE(R) ALL <1946 to September 10, 2024> |  |
| --- | --- | --- |
| 1 | [**Target Population**] |  |
| 2 | exp Glioma/ | 103595 |
| 3 | (glioma* or astrocytoma* or (astrocytic brain adj (tumor* or tumour* or cancer* or neoplasm*)) or astroblastoma* or ependymoma* or subependymoma* or craniopharyngioma* or oligodendroglioma* or glioblastoma* or GBM* or oligoastrocytoma* or xanthoastrocytoma* or ganglioglioma* or gangliocytoma* or gliosarcoma* or glioneuronal* or (glial adj2 (tumor* or tumour* or cancer* or neoplasm*))).mp. | 152910 |
| 4 | (brain adj (tumor* or tumour* or cancer* or neoplasm*)).tw,kf. | 67266 |
| 5 | (brain adj1 metasta*).tw,kf. | 20752 |
| 6 | ((cereb* or intracereb* or choroid plexus or crani* or intracrani* or intra-crani* or infratentori* or infra-tentori* or subtentori* or sub-tentori* or supratentori* or supra-tentori* or hypothalam* or pituitar*) adj3 metasta*).tw,kf. | 6741 |
| 7 | ((mening* or leptomening*) adj3 metasta*).tw,kf. | 3186 |
| 8 | ((CNS or central nervous system or spinal cord or epidur*) adj (tumor* or tumour* or cancer* or neoplasm*)).tw,kf. | 12018 |
| 9 | ((CNS or central nervous system or spinal cord or epidur*) adj3 metasta*).tw,kf. | 4418 |
| 10 | exp *central nervous system neoplasms/ or exp *brain neoplasms/ | 184183 |
| 11 | exp central nervous system neoplasms/di or exp brain neoplasms/di | 38761 |
| 12 | or/2-11 | 296774 |
| 13 | [**Liquid Biopsy (Urine/CFS)**] |  |
| 14 | Liquid Biopsy/ | 3149 |
| 15 | (liquid adj (biops* or biomarker* or bio-marker*)).tw,kf. | 10685 |
| 16 | Urinalysis/ | 9520 |
| 17 | (urine or urinary or urinalys*).tw,kf. | 569399 |
| 18 | Urine.fs. [fs: floating subheading] | 220672 |
| 19 | Cerebrospinal Fluid/ | 19665 |
| 20 | (CSF or cerebro-spinal fluid* or cerebrospinal fluid*).tw,kf. | 181414 |
| 21 | Cerebrospinal Fluid.fs. [fs: floating subheading] | 53953 |
| 22 | or/14-21 | 841945 |
| 23 | 12 and 22 [Target Population AND Liquid Biopsy] | 14105 |
| 24 | [**Key Biomarkers - identified from Search-1**] |  |
| 25 | circulating microrna/ [MeSH] | 1780 |
| 26 | (circulat* adj3 (microRNA* or micro-RNA* or miRNA* or mi-RNA* or miR or mi-R or miRs or mi-Rs)).tw,kf. | 7539 |
| 27 | (cell free adj3 (microRNA* or micro-RNA* or miRNA* or mi-RNA* or miR or mi-R or miRs or mi-Rs)).tw,kf. | 424 |
| 28 | ((extracellular or extra-cellular) adj3 (microRNA* or micro-RNA* or miRNA* or mi-RNA* or miR or mi-R or miRs or mi-Rs)).tw,kf. | 1975 |
| 29 | (exosom* adj3 (microRNA* or micro-RNA* or miRNA* or mi-RNA* or miR or mi-R or miRs or mi-Rs)).tw,kf. | 5945 |
| 30 | ((small adj3 temporal adj3 RNA) or stRNA* or st-RNA*).tw,kf. | 63 |
| 31 | or/25-30 | 14744 |
| 32 | 23 and 31 | 72 |
| 33 | MicroRNAs/cf, ur [Cerebrospinal Fluid, Urine] [MeSH + subheadings] | 545 |
| 34 | 12 and 33 | 29 |
| 35 | 32 or 34 | 90 |
| 36 | (microRNA21 or microRNA-21 or micro-RNA21 or micro-RNA-21 or miRNA21 or miRNA-21 or mi-RNA21 or mi-RNA-21 or miR21 or miR-21 or mi-R21 or mi-R-21).tw,kf. | 9379 |
| 37 | 23 and 36 | 34 |
| 38 | (microRNA15a* or microRNA-15a* or micro-RNA15a* or micro-RNA-15a* or miRNA15a* or miRNA-15a* or mi-RNA15a* or mi-RNA-15a* or miR15a* or miR-15a* or mi-R15a* or mi-R-15a*).tw,kf. | 1164 |
| 39 | 23 and 38 | 1 |
| 40 | (microRNA15b* or microRNA-15b* or micro-RNA15b* or micro-RNA-15b* or miRNA15b* or miRNA-15b* or mi-RNA15b* or mi-RNA-15b* or miR15b* or miR-15b* or mi-R15b* or mi-R-15b*).tw,kf. | 780 |
| 41 | 23 and 40 | 3 |
| 42 | (microRNA16* or microRNA-16* or micro-RNA16* or micro-RNA-16* or miRNA16* or miRNA-16* or mi-RNA16* or mi-RNA-16* or miR16* or miR-16* or mi-R16* or mi-R-16*).tw,kf. | 3640 |
| 43 | 23 and 42 | 3 |
| 44 | (microRNA19b* or microRNA-19b* or micro-RNA19b* or micro-RNA-19b* or miRNA19b* or miRNA-19b* or mi-RNA19b* or mi-RNA-19b* or miR19b* or miR-19b* or mi-R19b* or mi-R-19b*).tw,kf. | 805 |
| 45 | 23 and 44 | 3 |
| 46 | (microRNA24* or microRNA-24* or micro-RNA24* or micro-RNA-24* or miRNA24* or miRNA-24* or mi-RNA24* or mi-RNA-24* or miR24* or miR-24* or mi-R24* or mi-R-24*).tw,kf. | 1353 |
| 47 | 23 and 46 | 2 |
| 48 | (microRNA92* or microRNA-92* or micro-RNA92* or micro-RNA-92* or miRNA92* or miRNA-92* or mi-RNA92* or mi-RNA-92* or miR92* or miR-92* or mi-R92* or mi-R-92*).tw,kf. | 1752 |
| 49 | 23 and 48 | 5 |
| 50 | (microRNA106b* or microRNA-106b* or micro-RNA106b* or micro-RNA-106b* or miRNA106b* or miRNA-106b* or mi-RNA106b* or mi-RNA-106b* or miR106b* or miR-106b* or mi-R106b* or mi-R-106b*).tw,kf. | 875 |
| 51 | 23 and 50 | 2 |
| 52 | (microRNA155* or microRNA-155* or micro-RNA155* or micro-RNA-155* or miRNA155* or miRNA-155* or mi-RNA155* or mi-RNA-155* or miR155* or miR-155* or mi-R155* or mi-R-155*).tw,kf. | 5456 |
| 53 | 23 and 52 | 1 |
| 54 | (microRNA204* or microRNA-204* or micro-RNA204* or micro-RNA-204* or miRNA204* or miRNA-204* or mi-RNA204* or mi-RNA-204* or miR204* or miR-204* or mi-R204* or mi-R-204*).tw,kf. | 1136 |
| 55 | 23 and 54 | 1 |
| 56 | (microRNA10b* or microRNA-10b* or micro-RNA10b* or micro-RNA-10b* or miRNA10b* or miRNA-10b* or mi-RNA10b* or mi-RNA-10b* or miR10b* or miR-10b* or mi-R10b* or mi-R-10b*).tw,kf. | 949 |
| 57 | 23 and 56 | 3 |
| 58 | (microRNA218* or microRNA-218* or micro-RNA218* or micro-RNA-218* or miRNA218* or miRNA-218* or mi-RNA218* or mi-RNA-218* or miR218* or miR-218* or mi-R218* or mi-R-218*).tw,kf. | 888 |
| 59 | 23 and 58 | 0 |
| 60 | (microRNA193b* or microRNA-193b* or micro-RNA193b* or micro-RNA-193b* or miRNA193b* or miRNA-193b* or mi-RNA193b* or mi-RNA-193b* or miR193b* or miR-193b* or mi-R193b* or mi-R-193b*).tw,kf. | 453 |
| 61 | 23 and 60 | 0 |
| 62 | (microRNA331* or microRNA-331* or micro-RNA331* or micro-RNA-331* or miRNA331* or miRNA-331* or mi-RNA331* or mi-RNA-331* or miR331* or miR-331* or mi-R331* or mi-R-331*).tw,kf. | 242 |
| 63 | 23 and 62 | 0 |
| 64 | (microRNA374a* or microRNA-374a* or micro-RNA374a* or micro-RNA-374a* or miRNA374a* or miRNA-374a* or mi-RNA374a* or mi-RNA-374a* or miR374a* or miR-374a* or mi-R374a* or mi-R-374a*).tw,kf. | 215 |
| 65 | 23 and 64 | 0 |
| 66 | (microRNA548c* or microRNA-548c* or micro-RNA548c* or micro-RNA-548c* or miRNA548c* or miRNA-548c* or mi-RNA548c* or mi-RNA-548c* or miR548c* or miR-548c* or mi-R548c* or mi-R-548c*).tw,kf. | 69 |
| 67 | 23 and 66 | 0 |
| 68 | (microRNA520f* or microRNA-520f* or micro-RNA520f* or micro-RNA-520f* or miRNA520f* or miRNA-520f* or mi-RNA520f* or mi-RNA-520f* or miR520f* or miR-520f* or mi-R520f* or mi-R-520f*).tw,kf. | 37 |
| 69 | 23 and 68 | 0 |
| 70 | (microRNA27b* or microRNA-27b* or micro-RNA27b* or micro-RNA-27b* or miRNA27b* or miRNA-27b* or mi-RNA27b* or mi-RNA-27b* or miR27b* or miR-27b* or mi-R27b* or mi-R-27b*).tw,kf. | 832 |
| 71 | 23 and 70 | 0 |
| 72 | (microRNA130b* or microRNA-130b* or micro-RNA130b* or micro-RNA-130b* or miRNA130b* or miRNA-130b* or mi-RNA130b* or mi-RNA-130b* or miR130b* or miR-130b* or mi-R130b* or mi-R-130b*).tw,kf. | 518 |
| 73 | 23 and 72 | 0 |
| 74 | Fibroblast Growth Factor 2/cf, ur [Cerebrospinal Fluid, Urine]  [MeSH + subheadings] | 95 |
| 75 | 12 and 74 | 4 |
| 76 | (basic fibroblast growth factor or bFGF).tw,kf. | 17344 |
| 77 | 23 and 76 | 11 |
| 78 | 75 or 77 | 11 |
| 79 | Vascular Endothelial Growth Factor A/cf, ur [Cerebrospinal Fluid, Urine] [MeSH + subheadings] | 183 |
| 80 | 12 and 79 | 17 |
| 81 | (vascular endothelial growth factor or VEGF).ti. or ((vascular endothelial growth factor or VEGF) adj5 (biomarker* or bio-marker* or marker* or panel* or profil*)).ab,kf. [too sensitive if unqualified] | 32823 |
| 82 | 23 and 81 | 28 |
| 83 | 80 or 82 | 39 |
| 84 | Interleukin-8/cf, ur [Cerebrospinal Fluid, Urine]  [MeSH + subheadings] | 333 |
| 85 | 12 and 84 | 6 |
| 86 | (Interleukin8 or Interleukin-8 or IL8 or IL-8 or chemotactic factor* or chemotactic peptide* or cxcl8).tw,kf. | 62304 |
| 87 | 23 and 86 | 40 |
| 88 | 85 or 87 | 40 |
| 89 | Chemokine CCL2/cf, ur [Cerebrospinal Fluid, Urine] [MeSH + subheadings] | 390 |
| 90 | 12 and 89 | 7 |
| 91 | (CCL2* or (chemokine adj (ligand2* or ligand-2*))).tw,kf. | 17211 |
| 92 | 23 and 91 | 21 |
| 93 | 90 or 92 | 24 |
| 94 | Intercellular Adhesion Molecule-1/cf, ur [Cerebrospinal Fluid, Urine] [MeSH + subheadings] | 91 |
| 95 | 12 and 94 | 2 |
| 96 | (ICAM1 or ICAM-1 or ((intercellular or intra-cellular) adj adhesion adj (molecule1 or molecule-1)) or CD54 antigen*).tw,kf. | 28095 |
| 97 | 23 and 96 | 24 |
| 98 | 95 or 97 | 24 |
| 99 | Nogo Proteins/ [MeSH] | 894 |
| 100 | (NogoA or Nogo-A or ((isoformA or isoform-A) adj3 (reticulon4 or reticulon-4))).tw,kf. | 820 |
| 101 | 99 or 100 | 1165 |
| 102 | 23 and 101 | 3 |
| 103 | Myelin-Associated Glycoprotein/ and (Cerebrospinal Fluid or Urine).fs. [MeSH + subheadings] | 39 |
| 104 | 12 and 103 | 1 |
| 105 | (myelin-associated adj (protein* or glycoprotein)).tw,kf. | 1829 |
| 106 | 23 and 105 | 2 |
| 107 | 104 or 106 | 2 |
| 108 | ((oligodendrocyt* adj3 myelin adj3 glycoprotein) or OMGP).tw,kf. | 4158 |
| 109 | 23 and 108 | 5 |
| 110 | Nerve Tissue Proteins/ and (Cerebrospinal Fluid or Urine).fs. [MeSH + subheadings] | 673 |
| 111 | 12 and 110 | 16 |
| 112 | Neudesin*.tw,kf. | 38 |
| 113 | 23 and 112 | 1 |
| 114 | 111 or 113 | 16 |
| 115 | metabolom*.ti. or (metabolom* adj5 (biomarker* or bio-marker* or marker* or panel* or profil*)).tw,kf. [too sensitive if unqualified] | 33915 |
| 116 | 23 and 115 | 26 |
| 117 | ((cfDNA or ((cell free or circulat* or exosom* or extracellular or extra-cellular) adj3 DNA)) and fragment*).tw,kf. [urine fragmentation] | 1539 |
| 118 | 23 and 117 | 15 |
| 119 | ((WGS or ((whole genome* or exom*) adj3 sequenc*)) and (cfDNA or ((cell free or circulat* or exom* or exosom* or extracellular or extra-cellular) adj3 DNA))).mp. | 1161 |
| 120 | 23 and 119 | 28 |
| **121** | 35 or 37 or 39 or 41 or 43 or 45 or 47 or 49 or 51 or 53 or 55 or 57 or 78 or 83 or 88 or 93 or 98 or 102 or 107 or 109 or 114 or 116 or 118 or 120 | 322 |
|  | | |
| 122 | [**Key Biomarkers - zero hits above** - when limited to liquid biopsy, so combine with target population only (see line 124 below), then if too many hits limit to biomarker or diagnostic/prognostic filter, to define the context (see line 164) |  |
| 123 | 58 or 60 or 62 or 64 or 66 or 68 or 70 or 72 | 3182 |
| 124 | 12 and 123 | 124 |
| **Filters** (applied only if necessary) | | |
| 125 | (biomarker* or bio-marker* or ((tumor* or tumour* or cancer* or neoplas* or metasta*) adj5 (marker* or indicator*))).mp. | 886689 |
| 126 | [Diagnostic/Prognostic Filter] | 0 |
| 127 | Diagnosis/ | 17554 |
| 128 | Diagnosis, Differential/ | 472588 |
| 129 | "Diagnostic Techniques and Procedures"/ | 3748 |
| 130 | Diagnostic Test Approval/ | 113 |
| 131 | Diagnostic Tests, Routine/ | 15367 |
| 132 | Molecular Diagnostic Techniques/ | 14315 |
| 133 | (diagnos* adj3 (analys* or assay* or immunoassay* or classif* or differenti* or method* or kit or kits or panel? or predict* or screen* or system* or technique* or test*)).ab. | 447598 |
| 134 | diagnos*.ti,kf,hw,fx. | 4774552 |
| 135 | (diagnos* adj2 (performance or value?)).ab. | 71765 |
| 136 | (DTA or (diagnos* adj2 accura*)).tw,kf. | 129116 |
| 137 | "sensitivity and specificity"/ or "predictive value of tests"/ or roc curve/ or signal-to-noise ratio/ or "limit of detection"/ | 665468 |
| 138 | (sensitivity or specificity).tw,kf. | 1340960 |
| 139 | likelihood ratio*.tw,kf. | 21193 |
| 140 | (predict* adj4 val*).tw,kf. or predict*.ti. | 666918 |
| 141 | (prognos* adj4 val*).tw,kf. or prognos*.ti. | 270543 |
| 142 | ((re-test or retest or test-retest) adj reliability).tw,kf. | 26690 |
| 143 | ((accura* or reliab* or valid*) and (analys* or assay* or immunoassay* or classif* or detect* or diagnos* or differenti* or predict* or technique* or test*)).tw,kf. | 1994794 |
| 144 | Validation Study/ | 112245 |
| 145 | (validat* or validity).tw,kf. | 1011537 |
| 146 | area under curve/ | 46668 |
| 147 | observer variation/ | 45548 |
| 148 | (observer adj variation*).tw,kf. | 1781 |
| 149 | ((degree? or rate* or rating) adj3 agreement?).tw,kf. | 13310 |
| 150 | ((detect* or diagnos*) and agreement?).tw,kf. | 89332 |
| 151 | Receiver Operating Characteristic/ | 74831 |
| 152 | (receiver operating characteristic* or ROC).tw,kf. | 172429 |
| 153 | likelihood functions/ | 24070 |
| 154 | diagnostic error/ or false negative result/ or false positive result/ or missed diagnosis/ or false negative reactions/ or false positive reactions/ | 79320 |
| 155 | (false adj (positiv* or negativ*)).tw,kf. | 95359 |
| 156 | (QUADAS* or STARD).mp. | 4246 |
| 157 | laboratory diagnosis/ | 24025 |
| 158 | (reference standard? or gold standard?).tw,kf. | 123548 |
| 159 | (prognosis or progression or recurrence).hw. | 1089967 |
| 160 | (predict* adj3 (progressi* or recurren* or surviv*)).tw,kf. | 95718 |
| 161 | progression free survival.mp. | 78689 |
| 162 | (cut-off value or detection or recurrence or survival analysis).mp. | 1995568 |
| 163 | or/127-162 | 9340895 |
| 164 | 124 and (125 or 163) | 57 |
| Other types of key biomarker: | | |
| 165 | (extracellular vesicles/ or exosomes/) and (Cerebrospinal Fluid or Urine).fs. | 537 |
| 166 | 23 and 165 | 24 |
| 167 | ((extracellular or extracellular) adj3 vesicle*).ti. or (((extracellular or extracellular) adj3 vesicle*) and (biomarker* or bio-marker* or marker* or panel* or profil*)).ab,kf. [too sensitive if unqualified] | 19661 |
| 168 | 167 and 23 and (125 and 163) | 100 |
| 169 | 166 or 168 | 115 |
| 170 | proteomic*.ti. or (proteomic* adj5 (biomarker* or bio-marker* or marker* or panel* or profil*)).tw,kf. [too sensitive if unqualified] | 50659 |
| 171 | 170 and 23 and (125 and 163) | 40 |
| 172 | 121 or 164 or 169 or 171 | 494 |
| 173 | exp animals/ not humans/ | 5257232 |
| 174 | ((animal model* or mouse or mice or murine* or rat or rats or rodent* or muridae or murids or rabbit* or leporine* or leporidae or guineapig* or cavies or caviidae or hamster* or cricetidae or gerbil* or gerbillinae or cat or cats or feline* or felidae or dog or dogs or canine* or canidae or pig or pigs or piglet* or minipig* or swine* or porcine* or suidae or horse or horses or donkey or donkies or burros or asses or equine* or equidae or sheep or lamb or lambs or ovine or ovidae or goat or goats or cow or cows or cattle or bovine* or bovidae or primate* or monkey or monkeys or macaque or macaques or marmoset or marmosets) not human*).ti. | 2488768 |
| 175 | 173 or 174 | 5674389 |
| 176 | **172** not **175** | 461 |
| 177 | **Records not already retrieved by Search 1** | 252 |
